# Cytotoxic CD8^+^ T cells may be drivers of tissue destruction in Sjögren’s syndrome

**DOI:** 10.1038/s41598-022-19397-w

**Published:** 2022-09-14

**Authors:** Naoki Kaneko, Hu Chen, Cory A. Perugino, Takashi Maehara, Ryusuke Munemura, Shiho Yokomizo, Junsei Sameshima, Thomas J. Diefenbach, Katherine R. Premo, Akira Chinju, Yuka Miyahara, Mizuki Sakamoto, Masafumi Moriyama, John H. Stone, Seiji Nakamura, Shiv Pillai

**Affiliations:** 1 Ragon Institute of MGH, MIT and Harvard, 400 Tech Square, Cambridge, MA 02139 USA; 2 Section of Oral and Maxillofacial Oncology, Division of Maxillofacial Diagnostic and Surgical Sciences, Faculty of Dental Science, Kyushu University, Fukuoka, Japan; 3 OBT Research Center, Faculty of Dental Science, Kyushu University, Fukuoka, Japan; 4 Division of Rheumatology, Allergy, & Immunology, Massachusetts General Hospital, Harvard Medical School, Boston, MA USA

**Keywords:** Immunology, Diseases, Pathogenesis, Rheumatology

## Abstract

Sjögren’s syndrome is a chronic autoimmune disorder whose pathogenesis is poorly understood and that lacks effective therapies. Detailed quantitative and spatial analyses of tissues affected by Sjögren’s syndrome were undertaken, including the quantitation of the frequency of selected cell–cell interactions in the disease milieu. Quantitative analyses of CD4^+^ T cell subsets and of CD8^+^ T cells in the labial salivary glands from untreated patients with primary Sjögren’s syndrome revealed that activated CD8^+^ cytotoxic T cells (CD8^+^CTLs) were the most prominent T cells in these infiltrates. An accumulation of apoptotic glandular epithelial cells, mainly ductal and acinar cells, was observed, consistent with the impaired salivary secretion often observed in patients with this disease. FasL expressing activated CD8^+^ T cells were seen to accumulate around Fas expressing apoptotic epithelial cells. Quantitative analyses of apoptotic cell types and of conjugates between cytotoxic T cells and epithelial cells undergoing apoptosis suggest that Sjögren’s syndrome is primarily driven by CD8^+^CTL mediated execution of epithelial cells mainly represented by ductal and acinar cells.

## Introduction

Sjögren’s syndrome (SS) is an autoimmune disease characterized by the lymphocytic infiltration of affected glands, the concomitant destruction of glandular tissue and autoantibody production^1^. Primary Sjögren’s syndrome (pSS) is characterized by the presence of chronic exocrine glandular dysfunction in conjunction with serologic or histopathologic evidence of autoimmunity in the absence of other systemic rheumatologic diseases^1^. Reports on the use of systemic immunosuppression have inconsistently shown improvement in sicca symptoms, which may reflect the absence of a clear understanding of the pathogenesis of this disease^1,2^. A better understanding of the pathogenesis of any inflammatory disease with tissue involvement is likely to be best obtained by a detailed and quantitative interrogation of disease tissues.

A role for B cell and/or auto-antibodies in SS pathogenesis has sometimes been assumed. For instance, antibodies against the SS-A or SS-B antigens, proteins that are found in the nucleus or the cytosol, are of diagnostic value, but may not have any pathogenic significance except possibly in a small subset of patients with hypocomplementemia^3^. Although several studies including randomized double-blind clinical trials examining the utility of B cell depletion therapy in SS have been performed, the effect for clinical improvement can still be disputable^4–8^.

In SS, Th1 cells, Th2 cells, Th17 cells, T follicular helper (Tfh) cells and CD8^+^ T cells have all been implicated in the pathogenic process^9–17^. Several studies have used quantitative approaches and reported pivotal roles of T cells in the pathogenesis of SS^17–21^. However, previous studies have not used broad interrogation of T cells including several CD4^+^ T cell subsets in disease lesions. Some studies have demonstrated tissue destruction in SS, but multicolor staining approaches and quantification to ascertain the potential immunological basis of this destruction has not yet been undertaken^22–25^.

We quantitated known CD4^+^ T cell subsets and CD8^+^ T cells in SS tissues and explored the mechanism of tissue destruction in the labial salivary gland (LSG) tissues from patients with SS. We show here that CD3^+^ T cells that accumulate in LSG biopsies of untreated patients with pSS outnumber those of infiltrating B cells. Among the tissue-infiltrating T cells activated CD8^+^ cytotoxic T cells (CD8^+^CTLs) most often dominated the immune cell infiltrate. In addition, we identified prominent apoptosis of epithelial cells represented by ductal and acinar cells, the accumulation of CD8^+^CTLs expressing FasL in the vicinity of the epithelial cells and the upregulation of Fas by apoptotic cells in tissues affected by pSS, suggesting that CD8^+^CTLs may be of pathogenic relevance in directing tissue damage in SS.

## Results

### The most prominent T cell population is CD8^+^CTLs in SS

**Figure 1:**
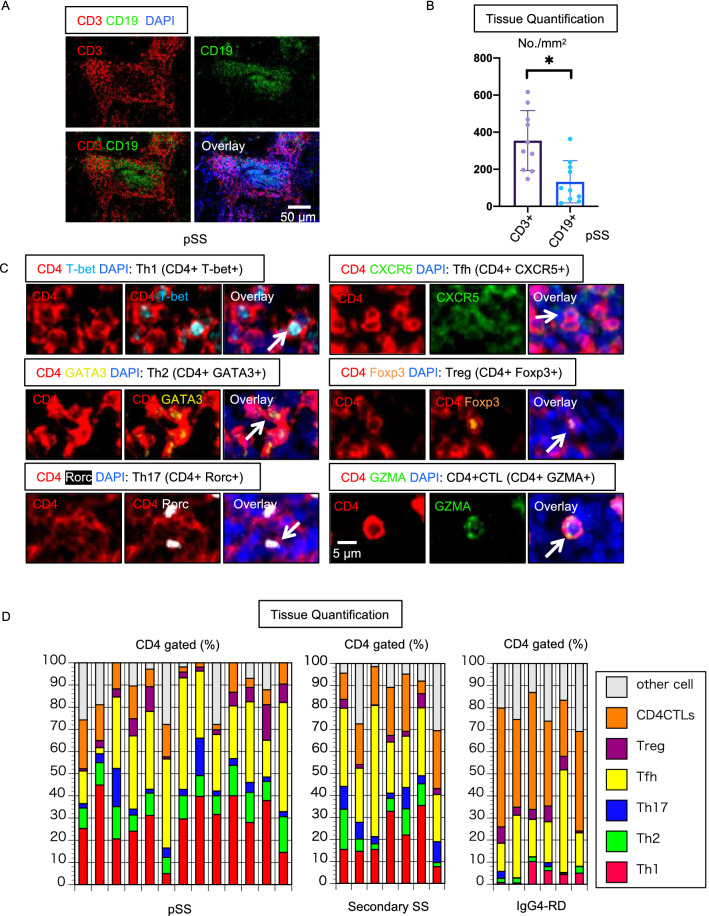
The most prominent CD4^+ ^T cell subsets in SS tissues are Th1 cells and Tfh cells. (**a**) Representative multi-color immunofluorescence image of CD3 (red), CD19 (green) and DAPI (blue) staining in a pSS lesion (patient number 2). (**b**) Absolute numbers of CD3^+^ T cells, CD19^+^ B cells in pSS (n = 10). Paired t test used to calculate p-value. (**c**) Representative multi-color staining showing each CD4^+^ T cell subset. [Th1: CD4+ (red) T-bet+ (light blue)] [Th2: CD4+ (red) GATA3+ (yellow)] [Th17: CD4+ (red) RORγ+ (white)] [Tfh: CD4+ (red) CXCR5+ (green)] [Treg: CD4+ (red) Foxp3+ (orange)] [CD4+ CTLs: CD4+ (red) GZMA+ (green)] (d) Relative proportions of Th1, Th2, Th17, Tfh, Treg and CD4+ CTL subsets in tissues from pSS (n = 13), secondary SS (n = 7) and IgG4-RD patients (n = 6). Error bars represent mean ± SEM. *p < 0.05.

In our analyses of the adaptive immune cells infiltrating tissues affected by SS, we concurrently quantitated the relative contribution of CD3^+^ T cells and CD19^+^ B cells in LSGs of 10 patients with pSS. While prominent numbers of both B and T cells were observed, the absolute number of infiltrating T cells was approximately twofold greater than those of infiltrating B cells (Fig. 1a,b). We focused on the T cells in the infiltrate across this group of patients given their relative preponderance in the tissue.

Given the varied literature regarding CD4^+^ T cell subsets in the context of SS, we first quantitated these subsets in the LSG tissues. In order to more quantitatively interrogate all major CD4^+^ T cell subsets, including Th1, Th2, Th17 and Tfh cells, as well as regulatory T (Treg) cells and CD4^+^ cytotoxic T cells (CD4^+^CTLs), we performed multi-color immunofluorescence by labeling T-bet, GATA3, RORγ, CXCR5, Foxp3, and GZMA on LSG tissues from 13 patients with pSS and seven patients with secondary SS. Representative images of markers used to define each CD4^+^ T cell subset are displayed in Fig. 1c. Since ICOS is considered another marker for Tfh cells, we also examined ICOS expression, and the majority of the CXCR5-positive cells were also positive for ICOS (Fig. S1). Although it is generally assumed that different pathogenetic processes drive pSS and secondary SS, CD4^+^ T cell subset distributions were strikingly similar when we compared tissues from primary and secondary SS patients^26^. In almost all the patients, Th1 and/or Tfh cells contributed most prominently to the overall CD4^+^ T cell infiltrate, in contrast to the very small proportion of Th17 or Treg cells (Fig. 1d). We examined salivary glandular tissues affected by IgG4-related disease (IgG4-RD) as an autoimmune disease control that also often affects the salivary glands^27^. Consistent with our previous reports implicating both CD4^+^CTLs and Tfh cells in the disease process of IgG4-RD, these two subsets accounted for the vast majority of CD4^+^ T cells whereas Th1 cells were relatively sparse^28,29^. The relative proportions of each CD4^+^ T cell subset in tissues from SS patients were clearly different from those in tissues affected by IgG4-RD (Fig. 1d).

**Figure 2:**
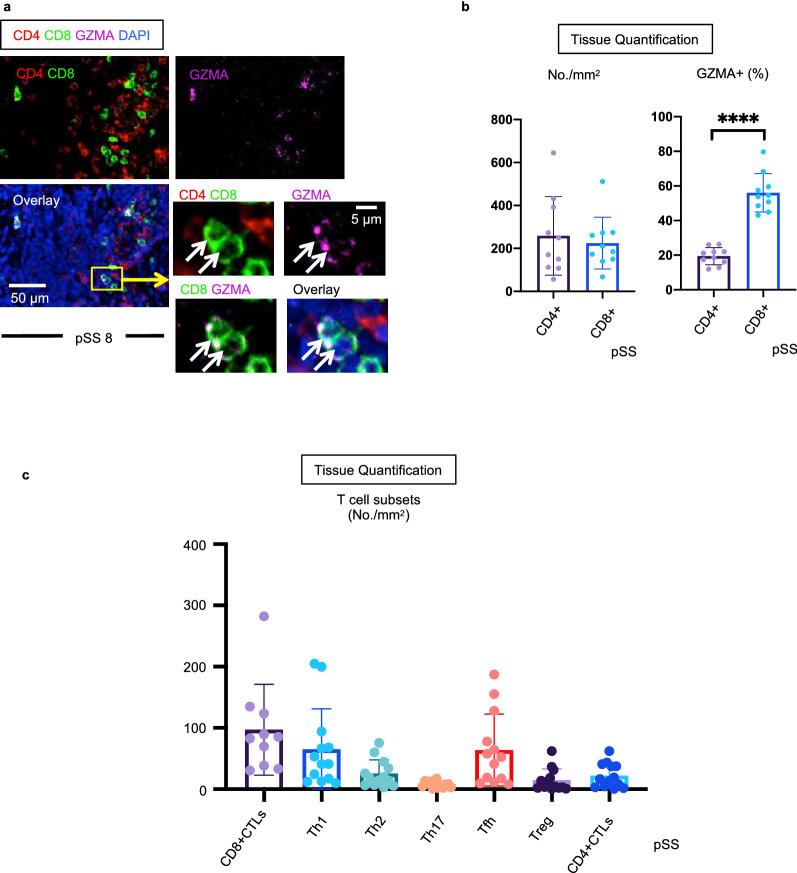
GZMA expressing CD8+ CTLs represent the most prominent T cell population in SS. (**a**) Representative multicolor immunofluorescence images showing CD4 (red), CD8 (green), Granzyme A (GZMA)(purple) and DAPI (blue) staining in pSS. White arrows indicate CD8^+^GZMA^+^ cells that have infiltrated the labial gland. (**b**) Left panel shows absolute numbers of CD4^+^ T cells and CD8^+^ T cells in pSS (n = 10). Right panel shows % of GZMA+ in CD4^+^ T cells and CD8^+^ T cells in pSS (n = 10). Paired t test used to calculate p-value. (**c**) Absolute numbers of each T cell subset (n = 13) and CD8^+^GZMA^+^ T cell (n = 10) in pSS. Error bars represent mean ± SEM. ****p < 0.0001.

To further explore the T cell infiltrate, we also quantitated CD8^+^ T cells in tissues from pSS patients. Although in theory only effector T cells enter tissue sites, we recognized that some more quiescent memory T cells could dominate these tissues as well^30^. Therefore, in order to quantitate fully differentiated CD8^+^CTLs we used granzyme-A (GZMA) as a marker of effector CD8^+^CTLs (Fig. 2a)^31^. The absolute number of CD8^+^ T cells was comparable to that of CD4^+^ T cells but the majority of GZMA expressing cells were CD8^+^ T cells with CD4^+^CTLs representing only a small portion of GZMA^+^ cells in pSS (Fig. 2b). The absolute numbers of CD8^+^CTLs were generally greater than those of any CD4^+^ T cell subset including Th1 and Tfh cells (Fig. 2c).

Other than CD4^+^CTLs and CD8^+^CTLs, NK cells are also cytotoxic when activated^32^. We stained tissues with antibodies to CD4, CD8 and NKp46, to identify CD4^−^CD8^−^NKp46^+^ NK cells (Fig. S2). CD4^+^ and CD8^+^ T cells infiltrated affected tissues in high numbers, but the numbers of infiltrating NK cells were negligible in pSS patients.

### Apoptotic cells are abundant in SS and IgG4-RD

**Figure 3:**
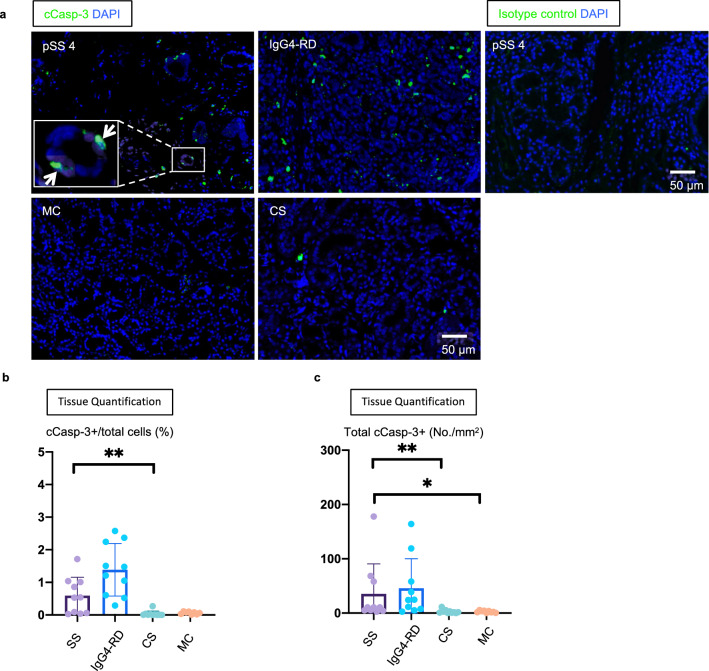
Apoptotic cells accumulate in tissues from SS and IgG4-RD patients. (**a**) Representative multi-color immunofluorescence images showing cleaved caspase-3 (cCasp-3) (green) and DAPI (blue) staining in pSS, IgG4-related disease (IgG4-RD), mucous cyst (MC) and chronic sialadenitis (CS). White arrows indicate cCasp-3 positive apoptotic cells. Right panel sowing isotype control (green) staining in pSS. (**b** and **c**) Absolute numbers (**b**) and proportions (**c**) of cCasp-3 positive apoptotic cells in pSS (n = 10), IgG4-RD (n = 10), MS (n = 8) and CS (n = 10). Multiple comparisons are controlled for by Dunn’s test. Error bars represent mean ± SEM. **p < 0.01; ***p < 0.001.

Given the relative abundance of GZMA^+^CD8^+^CTLs, we explored the possibility that these cells might induce the apoptotic death of specific cell types, presumably because the infiltrating CD8^+^ T cells were specific for an HLA class I molecule/self-peptide combination that could potentially reactivate these CD8^+^ T cells and thus induce them to kill their antigenic targets^33^. We identified apoptotic cells using cleaved caspase-3 (cCasp-3) as a marker of apoptosis and quantitated their frequency in LSG tissues from patients with pSS^34^. As controls we used IgG4-RD, mucous cysts (MC) and patients with chronic sialadenitis (CS). Although apoptotic cells are rapidly cleared in tissues by efferocytosis, we observed a higher frequency of apoptotic cells in pSS and IgG4-RD compared to MC and CS (Fig. 3a–c).

### Acinar cells and ductal cells might be potential targets of cytotoxicity in SS

**Figure 4:**
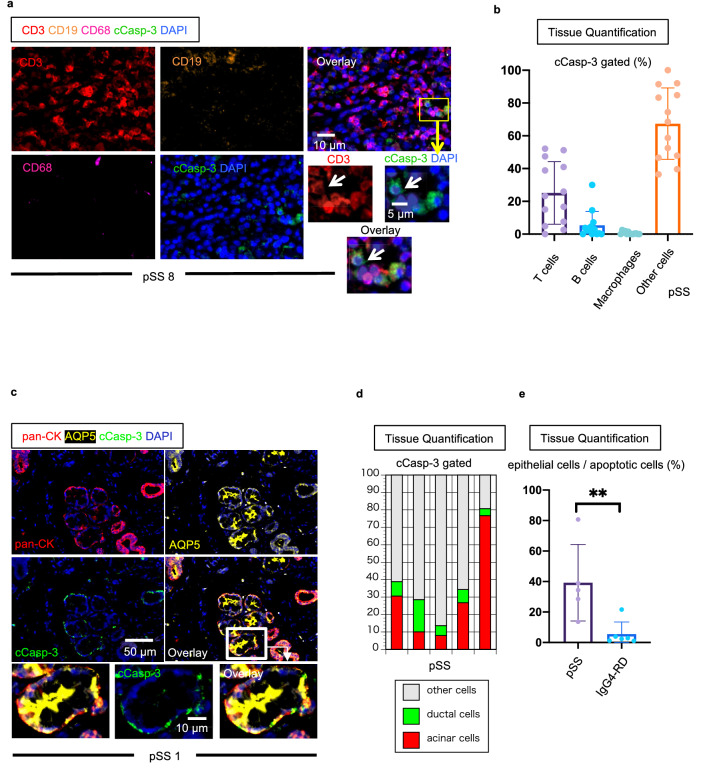
T cells, acinar cells and ductal cells account for a large proportion of apoptotic cells in tissues from SS patients. (**a**) Representative multi-color immunofluorescence image of CD3 (red), CD19 (orange), CD68 (purple), cCaps-3 (green) and DAPI (blue) staining in a pSS lesion. White arrows indicate apoptotic CD3^+^ T cells. (**b**) Proportions of apoptotic cells in pSS (n = 10) accounted for by T cells (red), B cells (green), Macrophages (blue) and other cells (gray). (**c**) Representative multi-color immunofluorescence images showing pan-CK (red), AQP5 (yellow), cCasp-3 (green) and DAPI (blue) staining in a pSS lesion. White arrows indicate a pan-CK^+^cCasp-3^+^ cell. (**d**) Proportions of apoptotic cells in pSS (n = 5) accounted for by acinar cells (red) (AQP5+, pan-CK+), ductal cells (green) (AQP5−, pan-CK+) and other cells (gray) (AQP5±, pan-CK−). (**e**) Relative proportions of cCasp-3^+^ cells expressing pan-CK or AQP5 or both in pSS (n = 5) and IgG4-RD (n = 6). Mann–Whitney U test used to calculate p-value. Error bars represent mean ± SEM. **p < 0.01; ***p < 0.001.

**Figure 5:**
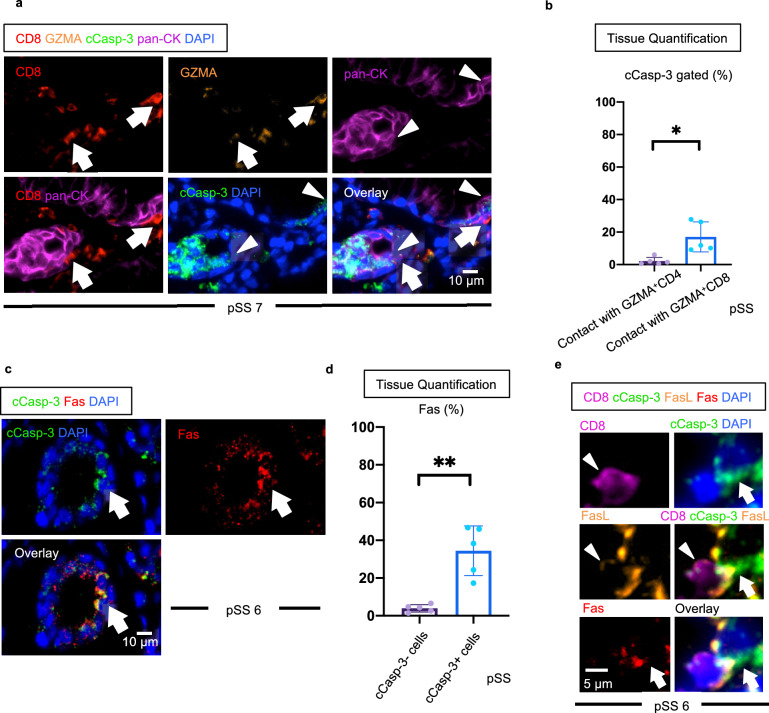
Epithelial cells expressing Fas are frequent targets of apoptosis in SS. (**a**) Representative multi-color immunofluorescence image showing GZMA (purple) expressing CD8^+^CTLs (white arrows) in close proximity to cCasp-3^+^ epithelial cells (arrow heads). (**b**) Relative proportions of cCasp-3+ cells in contact with CD4^+^ or CD8+ T cells. Paired t test was used to calculate p-value. (n = 5). (**c**) Multi-color immunofluorescence images of cCasp-3 (green), Fas (red) and DAPI (blue) staining. White arrows show cells co-expressing cCasp-3 (green) and Fas (red). (**d**) Relative proportions of cCasp-3−/+ cells expressing Fas (n = 5). Paired t test used to calculate p-value. (**e**) Multi-color immunofluorescence images show a CD8+ (purple) and FasL+ (orange) expressing cell in contact with a cCasp-3 (green) and Fas (red) expressing cell in a pSS tissue. Error bars represent mean ± SEM. **p < 0.01.

Several previous reports have described epithelial cell apoptosis in SS in a qualitative manner^22,23^. We quantitated apoptotic cells in pSS tissues and systematically attempted to identify the cell types that cCasp-3 was detected in. We initially co-stained for cCasp-3 along with CD3, CD19 and CD68 to broadly identify immune cells. Assessment of cCasp-3 expressing cells of immune origin demonstrated that a significant proportion of all apoptotic cells were CD3^+^ T cells, generally consistent with the expected activation induced cell death of effector T cells (Fig. 4a,b). Because the symptoms of dry mouth and dry eyes are suspected to be caused by the destruction of exocrine organs such as salivary glands and lacrimal glands, we then assessed the preferential targeting of acinar cells and ductal cells undergoing apoptosis by immunofluorescence using antibodies that recognize a broad array of keratins (pan-CK) and aquaporin 5 (AQP5) to define acinar (pan-CK^+^AQP5^+^) and ductal (pan-CK^+^AQP5^−^) cells in pSS tissue lesions^35–37^. We observed that a prominent proportion of apoptotic cells in pSS could be accounted for by dying acinar and ductal cells in most patients. In three of five cases, more than 30% of all apoptotic cells in pSS tissues were acinar or ductal cells (Fig. 4c,d). Subsequently, we examined apoptotic myoepithelial cells by examination of α-SMA, which is a marker for myoepithelial cells, and c-Casp3 staining in five pSS patients used in the manuscript since myoepithelial cells are also pan-CK positive. However, cCasp-3 and α-SMA staining did not overlap in all cases (Fig. S3). Although salivary glands are often inflamed in IgG4-RD patients, these patients generally do not suffer from dry mouth or diminished saliva production^38^. Consistent with this clinical difference, the relative proportions of acinar and ductal cell apoptosis were dramatically increased in pSS compared to IgG4-RD (Fig. 4e). We also examined the expression of HLA-DR in apoptotic cells in pSS, since CD4^+^CTLs are HLA class II restricted and we had previously reported that apoptotic cells in systemic sclerosis and IgG4-RD expressed HLA-DR^39,40^. In contrast to our observations in systemic sclerosis and IgG4-RD, we did not observe differences in HLA-DR expression between cCasp-3 positive cells and negative cells in pSS lesions (Fig. S4). These results and the relatively low proportion of CD4^+^CTLs in SS, suggest that CD4^+^CTLs are not important contributors to apoptotic cell death in this disease and support a more prominent role for CD8^+^CTL-mediated apoptotic elimination of antigenic cellular targets in pSS. Indeed, we often found CD8^+^CTLs in close proximity to apoptotic acinar and ductal cells with GZMA visible within the cytosol of the dying cells, suggesting that epithelial cells (pan-CK positive cells) are likely targets of CD8^+^CTLs (Fig. 5a). To further explore the possibility that CD8^+^CTLs contribute to tissue destruction in pSS, we quantitated the relative frequency of apoptotic cells in physical contact with either CD4^+^CTLs or CD8^+^CTLs. Apoptotic cell-CD8^+^CTL contacts were more frequently observed than apoptotic cell-CD4^+^CTLs contacts, supporting the view that CD8^+^CTLs, rather than CD4^+^CTLs, contribute to apoptotic cell death in this disease (Fig. 5b). Because CD8^+^CTLs can utilize both granule exocytosis and Fas signaling to execute target cells, we examined the expression of Fas in cCasp-3 positive cells in pSS lesions^41^. We observed a marked increase in the percentages of Fas expressing cells among all apoptotic cells compared with non-apoptotic cells (Fig. 5c,d). Furthermore, FasL expressing CD8^+^ T cells were frequently seen in contact with apoptotic cells that expressed Fas (Fig. 5e).

## Discussion

In our studies described here, quantitative interrogation of SS tissues has identified CD8^+^CTLs as the prominent adaptive immune cell that accumulates in this disease in addition to CD4^+^ helper T cells. Quantitative analyses of apoptotic cells revealed the accumulation of apoptotic epithelial cells represented by ductal and acinar cells in SS, and cell–cell interaction studies revealed the frequent proximity of GZMA^+^CD8^+^CTLs to their putative epithelial cell targets.

Broad interrogation of immune cells by quantitative analyses in SS tissues have never been undertaken previously, and our findings have attempted to elucidate the relevance of T cells, including several CD4^+^ T cell subsets, in this disease. Apart from CD8^+^ T cells, Th1 and Tfh cells were found to infiltrate tissues in high numbers but Th2 cells, Th17 cells, CD4^+^CTLs and Treg cells were not frequent. Th1 cells along with CD8^+^CTLs make up major components of a type I immune response and Th1 cells are also known to facilitate optimal cytotoxic T cell responses and T cell killing by secreting cytokines such as interferon-γ and IL-2^42,43^. These data argue that the disease may largely be driven by a type I immune response dominated by CD8^+^CTLs and the apoptotic killing of cellular self-targets. We also found a relative abundance of Tfh cells in SS tissues. This subset of T cells is known to facilitate T cell-dependent B cell responses^44^. These Tfh cell data are consistent with numerous previous reports documenting the production of anti-Ro/SS-A and anti-La/SS-B antibodies, the abundance of organ-specific and organ non-specific autoantibodies and rheumatoid factor, of circulating immune complexes, hypergammaglobulinemia, the formation of ectopic germinal centers, and reports of oligo-clonal B-cell proliferation in SS^1,45,46^. While a direct pathogenic role of autoantibodies is still questionable, B cells may also contribute to the pathogenesis of SS and should be more extensively interrogated in the future^21,47–51^.

**Figure 6:**
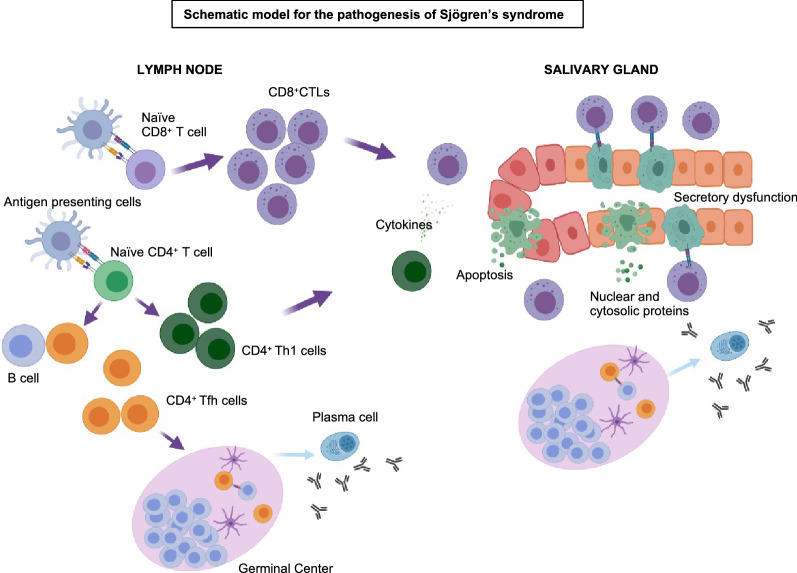
Schematic model for the pathogenesis of SS. Infiltration of tissues by Th1 CD4^+^ helper T cells and CD8^+^ T cells promotes inflammation, higher levels of Fas expression on epithelial (ductal/acinar) cells and CD8^+^ T cell mediated killing of acinar and ductal cell, resulting in irreversible secretory dysfunction. The antigenic targets of these CD8^+^ T cells are not known. The release of the contents of apoptotic cells including Ro/SS-A and La/SS-B and Tfh cells may help differentiation of B cells to plasma cells which produce antibodies to these autoantigens.

Overall, our data suggest that SS may be caused by self-reactive CD8^+^CTLs that induce the apoptotic cell death of ductal and acinar epithelial cells and thus contribute to the pathogenesis in collaboration with CD4^+^ helper T cells. A schematic overview of the pathogenesis of SS based on our observations is presented in Fig. 6. This potential disease mechanism would be consistent with the prominent secretory dysfunction observed in patients with SS. As expected, and as we have observed in other disorders^40^, a proportion of the effector T cells that infiltrate tissues also undergo apoptosis, consistent with existing knowledge that a large proportion of T cell effectors typically undergo contraction by activation-induced cell death. Our studies also suggest that apoptosis of ductal and acinar epithelial cells may occur via granule mediated cytotoxicity as well as by the Fas-FasL pathway. Granzyme A could theoretically also cause target cell death by pyroptosis, a phenomenon that we have not yet explored^31^. In any event, it is likely that the irreversible loss of ductal and acinar epithelial cells induced by activated CD8^+^CTLs results in the well-known secretory dysfunction of salivary and lacrimal glands in this disease. We speculate that cell death results in the release or discharge as apoptotic blebs of nuclear and cytosolic autoantigens such as SS-A and SS-B facilitating a secondary break in tolerance and autoantibody production^52^.

Qualitative reports have described apoptotic cells in other autoimmune diseases such as rheumatoid arthritis, systemic lupus erythematosus and multiple sclerosis^53–55^. We previously demonstrated the marked accumulation of apoptotic cells in the skin of patients with systemic sclerosis and most of the apoptotic cells were of endothelial origin, potentially contributing to the vasculopathy seen in that disease^39^. In IgG4-RD, we have also reported the accumulation of apoptotic cells in the affected tissues but in contrast to systemic sclerosis found more cells of mesenchymal origin as targets and very few apoptotic endothelial cells^41^. In contrast, the present study showed that a prominent proportion of apoptotic cells in pSS could be accounted for by epithelial cells including ductal and acinar cells. It is likely that different inciting auto-antigens and distinct immune mechanisms are relevant to each of these diseases. Future studies will focus on the identification of specific HLA class I restricted self-peptides recognized by CD8+ CTLs in SS. In addition, the limitation of this study is the cases we examined here are relatively advanced cases with lymphocytic infiltration. Therefore, it is possible that the patients in the early stage with less lymphocytic infiltration have a different overview of immunopathogenesis. Although several studies reported the pathogenetic roles of CD8+ T cells, the involvement of CD8+ T cells in disease initiation remains to be further explored^56–59^.

## Patients and methods

### Study participants

Labial salivary glands from 13 patients with pSS and seven patients with secondary SS, submandibular glands from ten patients with IgG4-RD and chronic sialadenitis, and labial salivary glands from eight patients with a mucous cyst were obtained through the Department of Oral and Maxillofacial Surgery of Kyushu University Hospital, Fukuoka, Japan. All patients had been evaluated between 1999 and 2018 at the Kyushu University Hospital^60^. Each patient with SS exhibited objective evidence of salivary gland involvement based on the presence of decreased salivary flow rate, abnormal findings on parotid sialography, and focal lymphocytic infiltrates in the LSGs by histology. All fulfilled the diagnostic criteria for definite SS proposed by the Research Committee on SS of the Ministry of Health and Welfare of the Japanese Government (1999)^61^, and the diagnosis was also based on the diagnostic criteria proposed by American College of Rheumatology (ACR)/European League Against Rheumatism (EULAR) classification criteria (2016)^62^. All patients with SS had severe disease and tertiary lymphoid organs were observed histologically in all patient samples. None of the patients with SS or IgG4-RD had current evidence of or a history of treatment with steroids or other immunosuppressants, infection with HIV, hepatitis B virus, hepatitis C virus, sarcoidosis, or lymphoma at the time of sample collection. Age, sex, serum Ig, and specific autoantibody levels, anti-SS-A/SS-B positivity, focus score, symptom duration, and saliva flow rate of patients with SS are summarized in Table S1. IgG4-RD was diagnosed as previously defined^27^. Chronic sialadenitis and mucous cysts were diagnosed based on clinical information. These studies were approved by the Institutional Review Boards at the Massachusetts General Hospital and Kyushu University Hospital. All patients provided written informed consent prior to inclusion in the study. The methods were carried out in accordance with the approved guidelines and regulations.

### Multi-color immunofluorescence staining

For multi-color immunofluorescence staining, tissue samples were fixed in formalin, embedded in paraffin, and 4 μm sectioned. The tissue sections were deparaffinized in xylene and rehydrated by serial passage through graded concentrations of ethanol. Endogenous peroxidase in tissues was blocked with 0.3% H_2_O_2_/methanol for 10 min. Heat-induced epitope retrieval was performed for 5 min at 95 °C in Tris–EDTA pH 9.0 buffer or Citrate pH 6.0 buffer. Sections were washed in cold running tap water and Tris-buffered saline–0.05% Tween20 (TBST) and blocked with blocking/antibody diluent for 10 min. Specimens were incubated with primary antibodies specific for the following proteins: anti-CD3 (dilution; 1:100) (clone: A045229-2; DAKO), anti-CD4 (1:250) (clone: CM153A; Biocare Medical), anti-CD19 (1:200) (clone: SKU310; Biocare Medical), anti-T-bet (1:500) (clone: ab150440; Abcam), GATA3 (1:800) (clone: CM405A; Biocare), Rorc (1:400) (clone: ab212496; Abcam), CXCR5 (1:400) (clone: MAB190; R&D Systems), Foxp3 (1:200) (clone: 98377; Cell Signaling Technology), anti-CD8 (1:2000) (clone: ab85792; Abcam), anti-CD68 (1:200) (clone: ab955; Abcam), anti-cleaved caspase-3 (1:400) (clone: 9664; Cell Signaling Technology), anti-GZMA (1:800) (clone: LS-C312742; LSBio), anti-pan-CK (1:200) (clone: ab27988; Abcam), anti-AQP5 (1:500) (clone: ab92320; Abcam), anti-HLA-DR (1:1000) (clone: ab20181; Abcam), anti-NKp46 (1:600) (clone: MAB1850; R&D Systems), anti-Fas (1:200) (clone: 4233; Cell Signaling Technology), anti-FasL (1:200) (clone: LS-B4195-50; LSBio), α-SMA (1:400) (clone: 19245; Cell Signaling Technology) and anti-ICOS (1:400) (clone: 89601S; Cell Signaling Technology). Then, sections were incubated with polymer HRP Mouse + Rabbit for 10 min, followed by incubation with an Opal fluorophore from Opal™ Multiplex Kit (Perkin Elmer) for 10 min. Bound primary and secondary antibodies were then eluted with heat-induced epitope retrieval treatment with stripping buffer. After washing in cold running tap water and TBST, the process of staining and antibody removal was repeated using a different Opal fluorophore^63^. Finally, the samples were mounted with ProLong™ Diamond Antifade mountant containing DAPI (Invitrogen).

### Microscopy and quantitative image analysis

Images of the tissue specimens were acquired using the TissueFAXS platform (TissueGnostics). For quantitative analysis, the entire area of the tissue was acquired as a digital grayscale image in five channels with filter settings for FITC, Cy3, Cy5 and AF75 in addition to DAPI. Cells of a given phenotype were identified and quantitated using the TissueQuest software (TissueGnostics), with cut-off values determined relative to the positive controls. This microscopy-based multicolor tissue cytometry software permits multicolor analysis of single cells within tissue sections similar to flow cytometry. StrataQuest software was used to quantify cell-to-cell contact. Overlap of at least 3 pixels of adjacent cell markers is required to establish a “contact” criterion. The principle of the method and the algorithms used have been described in detail elsewhere^64^.

### Statistical analyses

All statistical analyses were performed by GraphPad Prism version 8. A two-tailed Mann–Whitney *U*-test was used to calculate p-values for continuous, non-parametric variables. Paired t test was also performed to calculate p-values between two variables in the same subject. When comparing more than one population, Kruskal–Wallis testing was used with Dunn’s multiple comparison testing. In all analyses, p-values < 0.05 were considered statistically significant.

## Supplementary Information

Supplementary Information.

## Data Availability

The datasets used and analyzed during the current study are available from the corresponding author on reasonable request.
