# Supplementary material for: Cytotoxic CD8+ T cells may be drivers of tissue destruction in Sjögren’s syndrome
Source: Sci Rep. 2022 Sep 14;12:15427. doi: 10.1038/s41598-022-19397-w (PMC9475031; doi:10.1038/s41598-022-19397-w)
Supplement: Supplementary file 1 — Supplementary Information. [file 41598_2022_19397_MOESM1_ESM.pdf]

Supplementary Material

Cytotoxic CD8$^+$ T cells may be drivers of tissue destruction in Sjögren's syndrome

Naoki Kaneko[1,2,*], Hu Chen[2], Cory A Perugino[1,4], Takashi Maehara[2], Ryusuke Munemura[2], Shiho Yokomizo[2], Junsei Sameshima[2], Thomas J Diefenbach[1], Katherine R. Premo[1], Akira Chinju[2], Yuka Miyahara[2], Mizuki Sakamoto[2], Masafumi Moriyama[2,3], John H Stone[4], Seiji Nakamura[2] and Shiv Pillai[1,*]

[1]Ragon Institute of MGH, MIT and Harvard, 400 Tech Square, Cambridge, MA, USA

[2]Section of Oral and Maxillofacial Oncology, Division of Maxillofacial Diagnostic and Surgical Sciences, Faculty of Dental Science, Kyushu University, Fukuoka, Japan

[3]OBT Research Center, Faculty of Dental Science, Kyushu University, Fukuoka, Japan

[4]Division of Rheumatology, Allergy, & Immunology, Massachusetts General Hospital, Harvard Medical School, Boston, MA, USA

Contents of supplementary materials

Supplementary Table 1: Clinical data on Sjögren's syndrome patients studied

Supplementary Figure 1: Majority of CXCR5 expressing $CD4^+$ T cells are ICOS positive

Supplementary Figure 2: NK cells are relatively sparse in SS

Supplementary Figure 3: Apoptotic cells are not α-SMA positive cells represented by myoepithelial cells

Supplementary Figure 4: No difference in HLA-DR expression between apoptotic cells and non-apoptotic cells in pSS lesions

Table S1: Clinical data on Sjögren's syndrome patients studied

Age	Sex	Clinical Features	Serological Findings (mg/dL)						anti SS-A	anti SS-B	Focus Score	Symptom Duration	Salivary flow rate
		Past Medical History	IgG	IgA	IgM	IgE	RF	ANA			(Greenspan Grade)	(year)	Saxon test (g/min)
Primary SS													
82	F	-	2388	309	93	ND	ND	ND	-	-	3	-	2.73
70	F	Cirrhosis	3066	394	63	ND	44	40	+	+	7	3	1.81
57	F	-	1665	234	232	423	ND	40	-	-	4	-	1.51
58	F	-	3166	266	198	ND	65	1280	+	+	2	5	1.7
59	F	-	1380	189	120	＜10	ND	160	+	-	3	7	0.5
45	F	Anemia, Polyneuromyositis	1570	286	128	110	80	ND	+	-	5	2	0.95
30	F	-	1984	315	108		62	320	+	+	5	10	1.19
70	F	Cirrhosis	-	-	-	-	-	-	-	-	3	2	0.46
54	F	-	2203	254	214	ND	ND	ND	+	-	1	1	1.36
53	F	HTN, Appendicitis	1655	256	71	ND	62	160	+	+	9	1	0.17
76	F	Ovarian cyst, COPD, HTN	1645	256	332	ND	22	320	+	-	8	1	1.4
57	F	-	-	-	-	-	-	-	+	-	2	5	0.34
35	F	Cirrhosis, Ovarian	1715	166	157	ND	59	320	+	-	7	2	1.8
Secondary SS													
51	F	SSc	1773	436	84	ND	51	ND	-	-	3	3	1.03
48	F	AIP, DM, Renal angiomyolipoma	2607	239	32	ND	ND	ND	-	-	12	-	3.05
64	F	Rheumatoid arthritis, Galbladder	1688	317	81	ND	41	2560	+	+	4	-	0.35
48	F	Rheumatoid arthritis	2773	512	324	ND	614	80	+	+	10	-	4.67
66	F	AIP, DM, Fatty liver	1564	149	131	ND	ND	40	+	-	2	10	1.03
49	M	Rheumatoid arthritis, Thrombocytopeni	1191	282	120	ND	46	ND	+	+	3	9	1.52
66	F	AIP	1301	206	74	ND	ND	ND	-	-	7	2	4.33

Abbreviations: AIP, autoimmune pancreatitis; DM, diabetes mellitus; HTN, hypertension; COPD, chronic obstructive pulmonary disease

Supplementary Figure Legends

Supplementary Figure 1: Majority of CXCR5 expressing $CD4^+$ T cells are ICOS positive

(a) Representative multi-color immunofluorescence image of CD4 (red), ICOS (purple), CXCR5 (green), and DAPI (blue) staining in a pSS lesion. (b) Proportions of CD4+CXCR5+ T cells in pSS (n = 5) accounted for by ICOS+CXCR5+ dual positive Tfh cells (blue) and other cells (gold).

Supplementary Figure 2: NK cells are relatively sparse in SS

(a) Representative multi-color immunofluorescence image of CD4 (red), CD8 (green), NKp46 (purple) and DAPI (blue) staining in a pSS (upper) and an IgG4-RD (lower) lesion. (b) Absolute numbers of CD4 T cells, CD8 T cells and NK cells in pSS (n = 10) and IgG4-RD (n = 10). Multiple comparisons controlled for by Kruskal-Wallis test. Error bars represent mean ±SEM. **$p < 0.01$; ***$p < 0.001$.

Supplementary Figure 3: Apoptotic cells are not α-SMA positive cells represented by myoepithelial cells

(a) Representative multi-color immunofluorescence images of α-SMA (red), cCasp-3 (green) and DAPI (blue) staining in a pSS lesion. (b) Proportions of apoptotic cells in pSS (n = 5) accounted for by α-SMA positive cells including myoepithelial cells (red) and other cells (gray).

Supplementary Figure 4: No difference in HLA-DR expression between apoptotic cells and non-apoptotic cells in pSS lesions

(a) Representative multi-color immunofluorescence images of HLA-DR (red), cCasp-3 (green) and DAPI (blue) staining in a pSS lesion. (b) Percentages of HLA-DR+ cells in pSS (n = 5). Mann-Whitney U test used to calculate p-value.

Figure S1

a

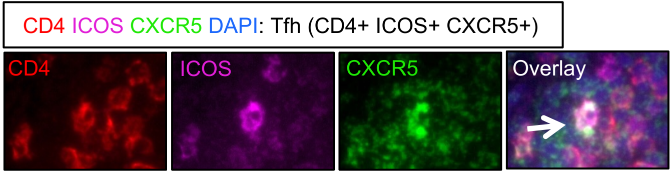

CD4 ICOS CXCR5 DAPI: Tfh (CD4+ ICOS+ CXCR5+)
CD4
ICOS
CXCR5
Overlay

b

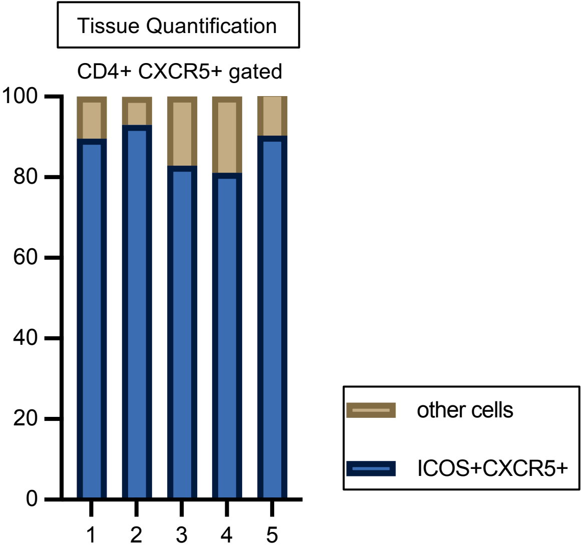

Tissue Quantification
CD4+ CXCR5+ gated
100
80
60
40
20
0
1
2
3
4
5
other cells
ICOS+CXCR5+

Figure S2

a

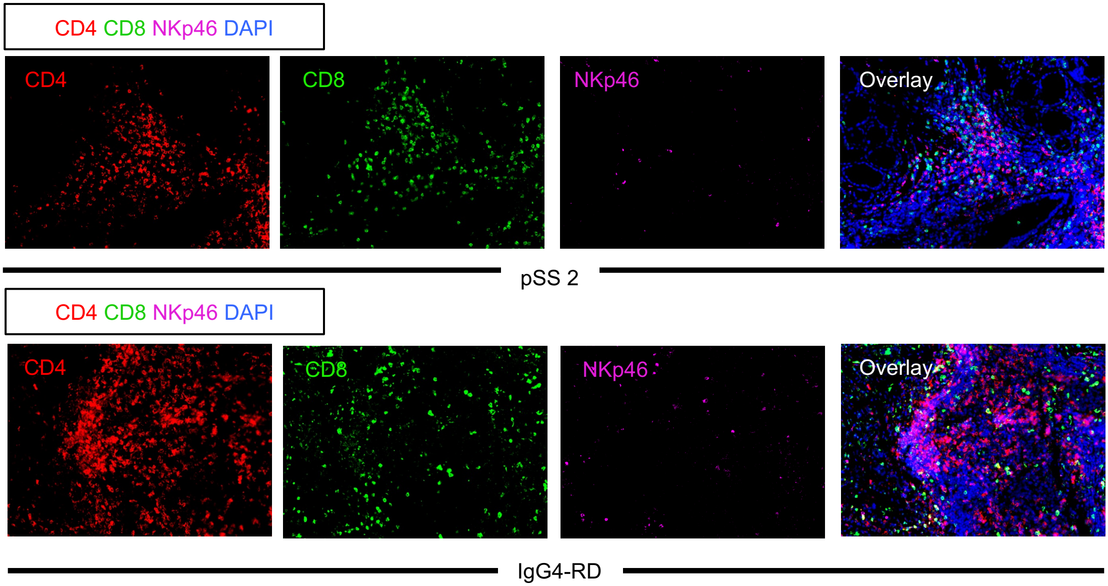
CD4 CD8 NKp46 DAPI
CD4
CD8
NKp46
Overlay
pSS 2
CD4 CD8 NKp46 DAPI
CD4
CD8
NKp46
Overlay
IgG4-RD

b

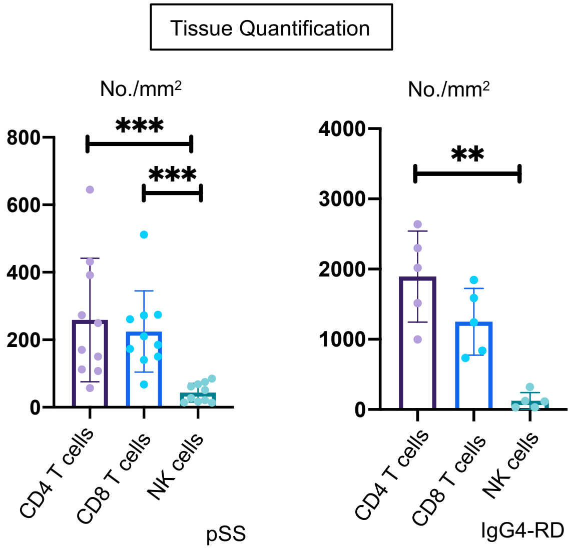
Tissue Quantification
No./mm2
800
600
400
200
0
***
***
CD4 T cells
CD8 T cells
NK cells
pSS
No./mm2
4000
3000
2000
1000
0
**
CD4 T cells
CD8 T cells
NK cells
IgG4-RD

Figure S3

a

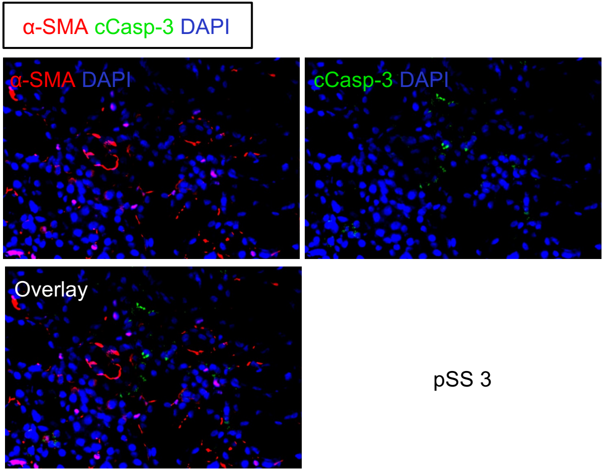

pSS 3

b

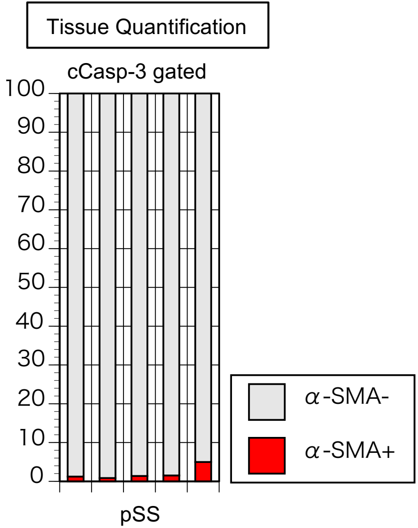

Figure S4

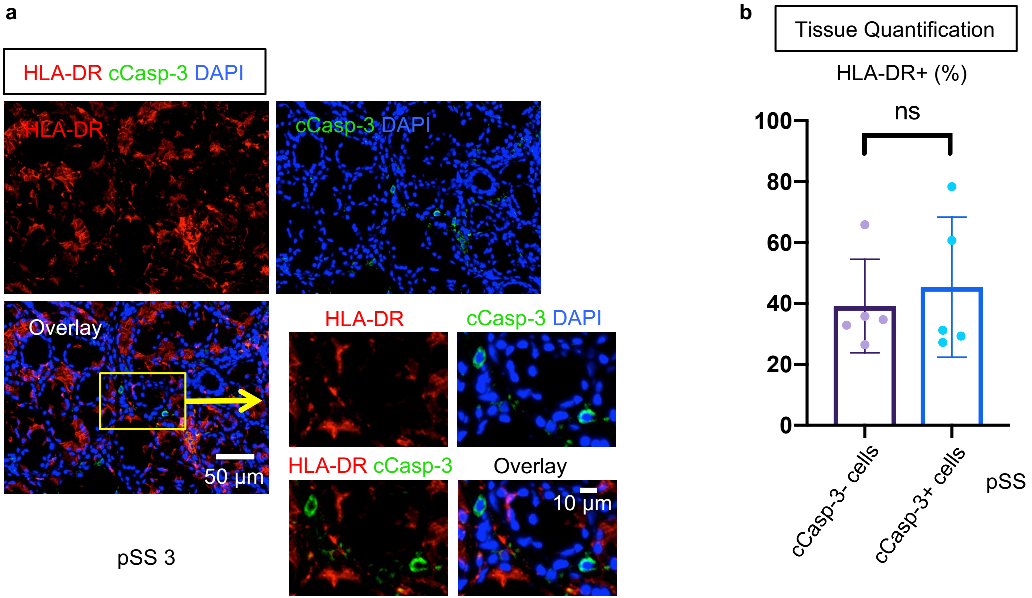
a
HLA-DR cCasp-3 DAPI
HLA-DR
cCasp-3 DAPI
Overlay
50 µm
HLA-DR
cCasp-3 DAPI
HLA-DR cCasp-3
Overlay
10 µm
pSS 3
b
Tissue Quantification
HLA-DR+ (%)
ns
100
80
60
40
20
0
cCasp-3- cells
cCasp-3+ cells
pSS
